# Can Population Mobility Make Cities More Resilient? Evidence from the Analysis of Baidu Migration Big Data in China

**DOI:** 10.3390/ijerph20010036

**Published:** 2022-12-20

**Authors:** Yu Chen, Keyang Li, Qian Zhou, Yuxin Zhang

**Affiliations:** 1 School of Economics and Management, Zhengzhou University of Light Industry, Science Avenue 136, Zhengzhou 450000, China; 2 Economics School, Zhongnan University of Economics and Law, Nanhu Avenue 182, Wuhan 430073, China; 3 Jingsh Lawyers Building, No. 37, East Forth Ring Middle Road, Beijing 100000, China

**Keywords:** population mobility, urban economic resilience, instrumental variable, Baidu migration big data

## Abstract

Knowledge spillover and capital agglomeration caused by population migration behavior are of great significance for improving the carrying capacity and adaptability of the urban economy and promoting high-quality economic development. Based on the big data collected on urban migration during the Spring Festival travel period, this paper constructs geographic, economic and geo-economic matrices, introduces two instrumental variables, and uses a spatial econometric model to investigate the mechanism between population mobility and urban economic resilience. The results show that (1) urban economic resilience exhibits spatial correlation, and the correlation order is geo-economic matrix > economic matrix > geography matrix; (2) the economic resilience of inflow areas is significantly affected by the net inflow of population, and the urban economic resilience index increases by 0.36–0.56% when the population mobility index increases by one unit; (3) in the case of economic and geo-economic matrices, there is a spatial interaction relationship of neighbor-companion in the mechanism of population migration on urban economic resilience; and (4) the mechanism is significantly impacted by innovation input and fixed asset investment, with positive moderating effects. In the geographical and economic matrices, the innovation input effect has a negative externality, while in the economic and geo-economic matrices, the fixed asset investment effect has a positive externality.

## 1. Introduction

Economic resilience is an important part of sustainable development research, as it is of great practical significance for expanding domestic demand, promoting the “double cycle”, upgrading industrial chains and high-quality economic development, and is currently a popular topic studied by Chinese and Western economic geographers [1]. As the core driver and an important carrier of economic development, the importance of cities in a country’s economic development cannot be overstated. How to help urban economies prevent and mitigate major risks has become the key to the formulation of economic policies in each country. Urban economic resilience refers to the ability of the urban economy, in the process of economic development, to cope with disturbances by constantly adjusting itself and learning from the disturbances [2,3]. Experience has shown that in the face of risks and challenges, if the city’s economic resilience is poor, the economic operation is more likely to be hit hard, resulting in the rapid decline of the city’s economy or even a collapse, seriously affecting social harmony and stability. With the outbreak of COVID-19 at the end of 2019, issues such as food security [4] and migration [5] under the background of the epidemic have attracted significant attention from the academic community, and public health has also been a hot topic of discussion [6]. In the context of the epidemic, public health is mainly caused by the movement of people. Therefore, in order to deal with the epidemic, countries have taken different measures to restrict the movements of the people, but this will undoubtedly bring another problem, that is, whether in terms of consumption or production, the economic development will be affected by the decrease of the flow of people. In this context, how do we enhance the ability of the urban economy to cope with risks? How can the city economy recover quickly from the crisis of population movement restriction? At present, there is no literature on the systematic elaboration of urban economic resilience from the perspective of population mobility. By sorting out the specific relationship between population mobility and urban economic resilience, and formulating corresponding measures according to the mechanism of action between the two, we can effectively ensure urban public health and minimize the impact of population mobility on economic resilience.

As the most populous country in the world, China’s population movement is also quite amazing. According to data, China’s mobile population reached 376 million in 2020; thus, it has become a social development phenomenon that cannot be ignored, especially the massive movement of the population during the Spring Festival, which does not occur in other parts of the world. Population mobility brings capital, information, material and technological flows to urban development, which is conducive to the agglomeration and dispersion of social and economic elements, as a result promoting the reallocation of production factors in the spatial field [7]. Statistics show that between 2010 and 2020, the GDP values of Guangdong, Zhejiang and Jiangsu, the three provinces with the largest population movements, grew by 144%, 137% and 151%, respectively. The literature mainly holds the following three viewpoints about the relationship between population flow and regional economic development: promotion, inhibition, and an inverted U-shaped nonlinear relationship [8,9,10]. However, few researchers have paid attention to the impact of population mobility on urban economic resilience. First, population migration helps to optimize resource allocation, improve labour productivity, and promote industrial structure upgrading [11], thus improving the tolerance and adaptability of urban economies to risks and enhancing urban economic resilience. Second, population migration is often accompanied by knowledge spillover effects, and an appropriate spatial concentration of the population will result in the accumulation of regional human capital, enrich the city’s knowledge base, enhance urban innovation, and make the transition more likely to be successful when risks arise.

In view of this, this research attempts to take population mobility as the entry point, construct a geography matrix, economy matrix, and geo-economic matrix, use the spatial lag (SAR) model, spatial error model (SEM) and spatial Dubin model (SDM), and introduce two instrumental variables, the similarity between dialect and Mandarin and the terrain up and down degrees, to construct a spatial model to explore the specific path to improve the economic resilience of Chinese cities. The discussion focuses on how migration affects urban economic resilience and how strong the impact is. In addition, insights into the innovation input effects and fixed asset investment effects of population mobility are revealed. In this paper, unless specified otherwise, population movement refers to the net inflow of the population.

The article is structured as follows: The second part reviews the relevant literature and develops hypotheses; the third part explains the variables and constructs the econometric model; the fourth part reports in detail the results of the empirical analysis of population mobility and urban economic resilience; the fifth part explores the moderating role of innovation inputs and fixed asset investment; and the sixth part provides the conclusion and suggestions.

## 2. Literature Review and Research Hypothesis

### 2.1. Population Mobility and Urban Economic Resilience

From a macro perspective, population migration helps to remove institutional barriers and reduce information costs, allows further opening to the world [12,13], and creates employment opportunities and income for the local market; furthermore, it is conducive to technological exchange and knowledge spillover between enterprises, enhancing the adaptability of the urban economy to external shocks [14] and bringing huge potential for economic development growth. In addition, a diversified industrial structure is one of the main ways to improve regional economic resilience development [15], especially when considering the characteristics of professional-diversified industrial allocation. A diversified industrial structure is the ideal state for regional economic resilience development [16], and industrial agglomeration relies on population agglomeration formed by population migration. From a micro perspective, population migration does not happen independently but is often accompanied by human capital and enterprise capital agglomeration. Human capital contains rich knowledge reserves and experience, which can help cities learn from disturbances and quickly adapt to the new state [17]. Corporate capital investment is conducive to the reorganization of capital factors in regional markets, and to some extent, it will also drive the reallocation of other factors, thus enhancing factor liquidity and improving the efficiency of resource allocation [18], which plays a very important role in cities’ responses to external crises and risks. At the same time, as the micro unit of urban economy resilient development, the diversification of knowledge and business reserves is conducive to helping the company withstand the unpredictable external market, and population mobility is the main way of forming this diversification. Therefore, this research proposes the following hypothesis:

**H1:**  *The population migration index is positively correlated with urban economic resilience.*

### 2.2. Population Mobility, Innovation Input and Urban Economic Resilience

Innovation input has the characteristics of a long cycle, high cost and great risk, especially under the general trend of China’s economic transformation and upgrading, and innovation input becomes more important. The mechanism of the effect of population mobility on urban economic resilience is impacted by innovation input. First, the knowledge spillover effect of population flow is more significant in regions with high innovation input, and it is easier to realize knowledge exchange and complete knowledge transfer [19,20] due to the introduction of high-level intellectuals or high-level technical personnel to these cities. Knowledge exchange between these individuals with high-end abilities and local personnel can inspire the urban initiative for innovation, as well as promote the innovation level. Innovation input plays a driving role in innovation activities and determines the technological innovation capability level [21]. Innovation capacity is the source of urban economic growth and industrial structure optimization, and it helps the urban economy resist external interference [16,22]. When the regional economy is impacted by external challenges and risks, the higher the innovation level of a city is, the more it can quickly adapt to the current situation, overcome obstacles, turn risks into opportunities and aid in economic recovery [23]. Second, cities with large investments in innovation are better able to cultivate innovative talent. Although the primary driver for development is innovation, “people” are the primary resource for development, and the most important input for innovation is the input of innovative talent. Population mobility brings a large number of human resources to urban areas, thus promoting economic development by strengthening urban innovation and creating a good innovation environment, which is conducive to transforming human resources into talent advantages to strengthen the interaction between talent and the economy and improving the ability of the urban economy to identify and respond to risks [24]. In addition, innovation activities also significantly depend on high-quality scientific and technological workers who have been trained, which is vital to improving the innovation level of a city [25]. Therefore, this research proposes the following hypothesis:

**H2:**  *Innovation input is the mechanism path between population mobility and urban economic resilience.*

### 2.3. Population Mobility, Fixed Asset Investment and Economic Resilience

Investment is considered to be one of the three main factors driving economic growth [26]. Since the 1990s, large-scale infrastructure construction dominated by government investment has become one of the main ways of developing China’s fixed capital, which has also greatly promoted China’s economic growth. Compared with the period of stable and healthy economic development, the recovery and development of the urban economy will be more dependent on fixed asset investment when faced with external uncertain risks and crisis challenges. The role of fixed asset investment in the impact of population flow on urban economic resilience can be summarized into the following three aspects. Firstly, from the perspective of resource support, fixed asset investment can not only improve people’s livelihood, but also play an important role in guiding the effective allocation of scarce resources and optimizing the industrial structure [27]. Increasing investment in fixed assets such as urban infrastructure construction can provide resource support for urban economy to cope with disturbances [28], enhance the ability of the urban economy to resist risks, and accelerate the recovery of the urban economy. Secondly, from the perspective of population inflow, the income gap is the major driver of population mobility [29]; however, studies show that fixed asset investment is also a major factor in promoting population inflow [30] because fixed asset investment represents the future economic development potential of cities. Areas with high urban fixed asset investment have great economic development potential and will attract a large population inflow, especially young floating talent. Finally, in terms of the effect on employment, reasonable fixed asset investment drives the formulation of industrial policies to a certain extent. By guiding relevant industries to flourish, a large labour demand is generated; thus, the labour force of the inflow cities is fully utilized and the demographic dividend is finally converted into a human capital dividend. Compared with cities with large population inflows but no high-quality employment, cities with scientific and reasonable industrial structures and employment guarantees are more likely to inject vitality into urban economic development and make full use of human resources. Therefore, the following hypothesis is proposed in this research.

**H3:**  *Fixed asset investment is the mechanism path between population mobility and urban economic resilience.*

**Figure 1:**
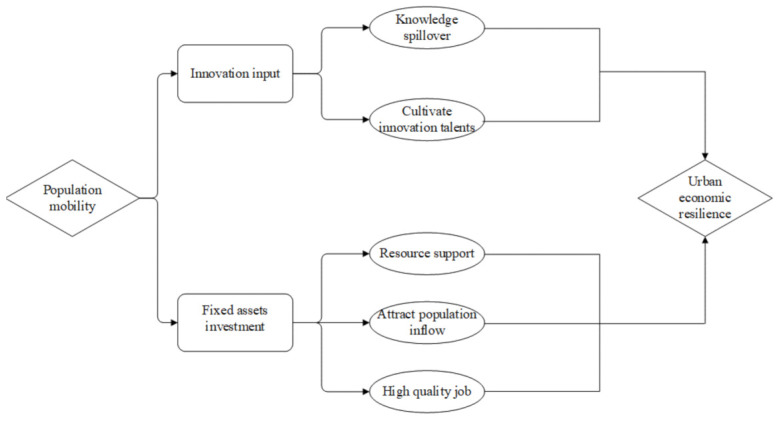
Population mobility and urban economic resilience.

In conclusion, the logical relationship between population flow and urban economic resilience is shown in Figure 1, that is, population flow can affect urban economic resilience through the knowledge spillover effect and cultivating innovation talents. In addition, fixed asset investment can also adjust the impact of population flow on urban economic resilience, mainly in terms of providing resource support for the urban economy, attracting population inflow and providing high-quality jobs.

### 2.4. Summary

Studies on the influencing factors of urban economic resilience mostly focus on the industrial structure [15], social culture [31], social capital [32], institutions and policies [33], and few papers take population mobility into account. The main contribution of this paper lies in the following: (1) Research theme: It enriches the existing research on the influencing factors of urban economic resilience and provides direct evidence for the relationship between population mobility and urban economic resilience; (2) Research data: Independent variable processing avoids the census data with static characteristics used in most studies. Based on Baidu migration big data, this paper more vividly describes the population mobility situation over a continuous period, which makes up for the limitation of the long period of census data and realizes an approximate estimation of long-term population migration; (3) Research methods: The lower the similarity between a local dialect and Mandarin, the lower the Mandarin proficiency of the local labor force, and the greater the possibility of language barriers in the communication between migrant workers and others, which will affect the population flow but basically not be affected by the resilience of the urban economy. Topographic relief can affect the change of population concentration and dispersion, but it does not affect the economic resilience of the city. Based on this, this paper introduces two instrumental variables, the similarity between Putonghua and dialect and topographic relief, and adopts the spatial regression model with instrumental variables to explore the relationship between population flow and urban economic resilience, which not only considers the spatial spillover effect, but also solves the endogeneity problem, and the results are more reliable.

## 3. Study Design, Variable Selection and Data Description

### 3.1. Study Design

#### 3.1.1. Entropy Method

This paper uses the entropy method to assign weight to the index system of urban economic resilience of prefecture-level cities in China, and calculates the crisis resistance index, urban recovery index and risk conversion index. The specific measurement steps can be roughly divided into three steps:

Firstly, according to the positive and negative of each index, the range method is used to standardize the original data.

(1) $$Positive\;\;\;indicators:\;\;\;{X_{ij}}^{\prime }=\frac{x_{ij}-\min(x_{ij})}{\max(x_{ij})-\min(x_{ij})}$$

(2) $$Negative\;indicators:\;\;\;{X_{ij}}^{\prime }=\frac{\max(x_{ij})-x_{ij}}{\max(x_{ij})-\min(x_{ij})}$$

where, $x_{ij}$ is the original value of the *i*th index in region *j*, ${X_{ij}}^{\prime }$ is the standardized value, $\max(x_{ij})$ and $\min(x_{ij})$ are its maximum and minimum values.

Secondly, the entropy of index $x_{ij}$ is calculated as follows:

(3) $$e_{j}=-k\sum_{i=1}^{n}p_{ij}\times \ln(p_{ij})$$

where, $pij=\frac{xij}{\sum_{i=1}^{n}xij}$, $K=1/ln\left(n\right)$, *n* is the number of samples, *e_j_* > 0.

Finally, calculate the economic resilience index $S_{i}$ of each city:

(4) $$S_{i}=\sum_{j=1}^{m}w_{j}\times x_{ij}$$

where, $W_{j}=\frac{d_{j}}{\sum_{j=1}^{m}d_{j}}$ is the weight of index *j*, $d_{j}=1-e_{j}$.

#### 3.1.2. Weight Matrix Setting

The spatial weight matrix should be constructed before performing spatial correlation analysis to objectively and comprehensively analyse the spatial spillover effect of urban economic resilience. This paper constructs geographical matrix $W_{ij}$, economic matrix $E_{ij}$, and their combination to form a geo-economic matrix $M_{ij}$ based on geographical distance features and socioeconomic features, respectively. The geographical matrix is based on the geographical distance between cities. Cities that are close in proximity are more closely connected than cities that are far from each other. The economic resilience of a city may be very likely affected by cities close to it.

(5) $$\begin{array}{l} W_{ij}=\left\{\begin{array}{l} \frac{1}{d_{ij}},\;d_{ij}\neq 0\\ 0,\;\;\;\;d_{ij}=0 \end{array}\right. \end{array}$$

(6) $$d_{ij}=\left\{\begin{array}{l} R\times ar\cos[\cos(Y_{i})\times \cos(Y_{j})\times \cos(X_{i}-X_{j})+\sin(Y_{i})\times \sin(Y_{j})],\;i\neq j\\ 0\;\;\;\;\;\;\;\;\;\;\;\;\;\;\;\;\;\;\;\;\;\;\;\;\;\;\;\;\;\;\;\;\;\;\;\;\;\;\;\;\;\;\;\;\;\;\;\;\;\;\;\;\;\;\;\;\;\;\;\;\;\;\;\;\;\;\;\;\;\;\;\;\;\;\;\;\;\;\;\;\;\;\;\;\;\;\;\;\;\;\;\;\;\;\;\;\;\;\;\;\;\;\;\;\;\;\;\;\;\;,i=j \end{array}\right.$$

In Formulas (5) and (6), $d_{ij}$ is the distance between city *i* and city *j*, R is the radius of the earth, $X_{i}$ the longitude of city *i*, and $Y_{i}$ is the dimension of city *i*.

The economic matrix is an indicator used to measure the “economic distance” between cities by using the balance of per capita GDP between cities. This is because the economic interactions between cities are not exactly the same. The higher the economic proximity between cities is, the shorter the “economic distance”.

(7) $$E_{ij}=\left\{\begin{array}{l} \frac{1}{\left|G_{i}-G_{j}\right|},i\neq j\\ 0\;\;\;\;\;\;\;\;\;\;\;,i=j \end{array}\right.$$

In Formula (7), $G_{i}$ is the per capita GDP of city *i* in 2019.

The geo-economic matrix introduces both geographical distance and “economic distance” into the spatial weight matrix to fit the economic relationship between adjacent areas.

#### 3.1.3. Spatial Correlation Test

A spatial correlation can reveal the spatial interactions among economic factors by discovering the spatial regional distribution characteristics of economic variables. When economic variables show regular nonrandom distributions in space, we usually assume that there is spatial correlation between the economic factors of different regions, which usually includes global spatial autocorrelation and local spatial autocorrelation.

Global spatial autocorrelation reflects the spatial similarity of attribute values of adjacent or close regions of observations in the whole research area. The global Moran’s I index is commonly used to calculate the correlation coefficient between observations and spatial lag variables.

(8) $$I=\frac{\sum_{i=1}^{n}\sum_{j=1}^{n}W_{ij}(X_{i}-\bar{X})(X_{j}-\bar{X})}{S^{2}\sum_{i=1}^{n}\sum_{j=1}^{n}W_{ij}}$$

In Formula (8), I is the global Moran’s I; n is the number of regions; $X_{i}$ and $X_{j}$ represent the urban economic resilience of region *i* and region *j*, respectively; and $W_{ij}$ is the spatial weight matrix.

Local spatial autocorrelation mainly analyses whether there are similar or different aggregation characteristics between the local area and surrounding area. Generally, Local Moran’s I, which is also a local indicator spatial association (LISA), is used to show which areas have similar aggregation and which areas have different aggregation, thus indicating the significance of the spatial difference degree.

(9) $$LISA=\frac{(X_{i}-\bar{X)}}{S_{X}^{2}}\sum_{i=1}^{n}\left[W_{ij}-(X_{i}-\bar{X)}\right]$$

In Formula (9), positive LISA, including H-H or L-L, indicates that the research unit is of high value and adjacent units are also of high value or that the research unit is of low value and adjacent units are also of low value. A negative LISA, including H-L or L-H, indicates that the research unit has a high value but the neighboring unit has a low value or that the research unit has a low value but the neighboring unit has a high value.

#### 3.1.4. Spatial Econometric Model

The same economic variable not only has spatial correlation in different regions but may also interact with other variables inside or outside the space, which is usually called the spatial effect. A spatial econometric model is generally used to measure the spatial effect of economic variables. At present, the commonly used spatial econometric models include SAR, SEM and SDM. The spatial lag model is used to analyse the spatial spillover effect of dependent variables, that is, the influence of dependent variables of neighbouring regions on local dependent variables. The spatial error model is used to consider the spatial spillover effect of unobservable factors and the influence of disturbance term factors in neighbouring regions on local dependent variables. The spatial Dubin model is an SAR model enhanced by adding the spatial lag term of independent variables, that is, the spatial spillover effect of dependent variables and independent variables is considered.

Anselin [34] provides the following general spatial cross section econometric model form:

(10) $$y=\rho W_{1}y+\beta X+\theta W_{2}X+\mu$$

(11) $$\mu =\lambda W_{3}\mu +\varepsilon$$

(12) $$\varepsilon \sim N(0,\sigma^{2}I_{n})$$

where y is the n-dimensional vector of the dependent variable, X is the independent variable matrix of the n × k dimension, and $W_{1}$, $W_{2}$, and $W_{3}$ are the spatial weight matrices of the n × n dimension, which can be the same matrix or different matrices. ρ is the spatial autocorrelation coefficient of the dependent variable, β is the relative parameter vector of the independent variable, θ is the spatial autocorrelation coefficient of the independent variable, λ is the spatial error coefficient, $I_{n}$ is the n-order identity matrix, and μ and ε are the random error terms.

(1) When ρ ≠ 0 and θ = 0, λ = 0, it is the SAR model;

(2) When λ ≠ 0 and ρ = 0, θ = 0, the model is SEM;

(3) When ρ ≠ 0, θ ≠ 0 and λ = 0, the model is SDM.

### 3.2. Variable Selection and Data Description

Taking 287 prefecture-level cities in China as the research object, the *China City Statistical Yearbook* and the statistical yearbook of provinces and cities are the major sources of data in this study. Of all the factors that affect the resilience of cities, population mobility is the main object of study. To overcome possible estimation errors, the urban size, level of economic openness, financial environment, government policies, infrastructure construction, information level and urban-rural income gap were selected as control variables, and the similarity between dialects and Mandarin and terrain up and down degrees were selected as instrumental variables. As moderating variables, innovation input and fixed asset investment are chosen to examine the mechanism pathway through which population mobility affects urban economic resilience.

#### 3.2.1. Explained Variable: Urban Economic Resilience (Resi)

Urban economic resilience includes the absorption, recovery as well as transformation of disturbance factors by the urban economy. It not only emphasizes the capacity to adapt to crises [35,36], but also focuses more on the capacity of the urban economy to transform itself after absorbing external disturbances into the economic system [3]. Therefore, this paper constructs a comprehensive assessment index system from the three dimensions of crisis resistance, urban resilience and risk transformation power on the basis of data availability (Table 1).

Crisis resistance points to the capacity of an urban economic system to resist or absorb these unfavorable factors when it encounters external disturbances to maintain its own equilibrium state and ensure normal operation of the urban economic system. The standard-level indicators include per capita GDP, import and export trade volume and the proportion of secondary and tertiary industry output value in GDP [37,38]. Urban resilience refers to the capacity of the urban economy when it encounters the impact of external risks to adapt to the new state quickly, resume production and construction and ensure that urban economic operation is not affected. The standard-level indicators select the per capita CNY deposit balance of financial institutions, per capita general public budget revenue and the proportion of employees in tertiary industry [39]. Risk transformation power refers to the ability to quickly switch from the previously broken equilibrium state to a more adaptive state when the urban economy is subject to greater external pressure and risk impacts, the normal operation of the urban economy is hindered, and restoring it to its original state is difficult. The urban economy can quickly switch from the previously damaged balance to a new balance that is more suitable for the current situation. The standard-level indicators include the proportion of expenditures on science and education, the number of college students per 10,000 people, and the total retail sales of consumer goods per capita [37].

#### 3.2.2. Core Explanatory Variable: Population Mobility (Mob)

Baidu is one of the four major AI companies in the world. It has a strong Internet foundation and rich products, which are represented by the Baidu search engine, Baidu Post Bar, Baidu Web disk and Baidu Map. Baidu Map is a new generation of maps based on artificial intelligence technology to provide customers with route planning, navigation and implementation of road conditions. The population mobility index data needed for the study were retrieved from the Baidu migration web page by using Python software, which is called Baidu Migration Big Data and is one of the products of the Baidu Map. The data is based on Baidu location-based service (LBS) technology. Through the location ware device, it can reflect the track with the space-time characteristic of daily population migration in a real-time, dynamic and visual way. The migration scale index can reflect the scale of people moving in or out of cities in China and can be used for horizontal comparison between cities.

Of all the festivals in China, the Spring Festival, which is the day for family reunion, is the most vital. Most people choose to return to their hometowns in the first two weeks of the Spring Festival and then return to work in the two weeks after the Spring Festival, which gives rise to the unique cultural phenomenon of Spring Festival transportation. The study period was two weeks after the Spring Festival, from 11 February 2019, to 24 February 2019. This period is the peak period for the floating population to migrate out for work, and most of them are those who are migrating out to work in other places and students who are going out to study. Therefore, the scale of population migration in the two weeks after the Spring Festival can approximately reflect the inflow and outflow of the population in each city in that year [40]. The specific measurement steps are as follows:

(13) $$N_{i}^{in}=\sum_{j=1}^{14}p_{ij}^{in}$$

(14) $$N_{i}^{out}=\sum_{j=1}^{14}p_{ij}^{out}$$

(15) $$Mob_{i}=\frac{N_{i}^{in}}{N_{i}^{out}}$$

where *i* represents the name of the city, *j* represents the *j*th day, and its value ranges from 1 to 14. $p_{ij}^{in}$ represents the population inflow index of city *i* on the *j*th day after the Spring Festival; $p_{ij}^{out}$ is the population outflow index of city *i* on the *j*th day; $N_{i}^{in}$ is the sum of the population inflow scale index of city *i*; and $N_{i}^{out}$ is the sum of the population outflow scale index of city *i*. $Mob_{i}$ represents the population flow index, and the value approximately represents the net population inflow of city *i* in 2019. $Mob_{i}$ is greater than 1, which means that city *i* is in the state of net inflow of population in that year. The larger the value is, the greater the net inflow of the population. $Mob_{i}$ is less than 1, which means that city *i* is in the state of net population outflow in that year. The smaller the value is, the greater the net outflow of the population.

#### 3.2.3. Control Variables

(1) Urban size (scal) is measured by the average annual population of a city. The expansion of the consumer market, the promotion effect of industrial agglomeration on the economy, and the ability of the urban economy to cope with risks are all aided by a modest growth in urban size [39].

(2) Economic openness (open) is the proportion of actual foreign capital utilized in GDP in that year is calculated. Keeping the economy open can not only create employment opportunities and increase income, but also facilitate technological exchange and knowledge spillover between enterprises, and increase the urban economy’s capacity to absorb external shocks, which will hasten the pace of its economic recovery [14,38].

(3) The financial environment (fina) is measured by financial efficiency. Finance is also a type of resource, and financial efficiency refers to the allocation efficiency of financial resources, which reflects the stability of the regional financial environment. However, it should be noted that higher financial efficiency is not better, and sometimes high financial efficiency can stifle economic development and reduce urban economic resilience [38].

(4) The government’s policy (gov) is measured by government spending as a percentage of GDP. Government policy can attract high-quality talent, promote technological innovation, and guide struggling industries. Public financial expenditure is an important aspect of government policy guidance, mainly through financial support behaviour to help affected enterprises recover development. Therefore, public financial expenditure is selected to reflect the regulatory capacity of a city to deal with risks [22,41].

(5) Infrastructure construction (infr) is measured by the actual urban road area in the city at the end of each year. It is the basis of urban economic growth; when the urban economy is disturbed by the outside, it can ensure that the basic functions of the city will not be affected.

(6) Level of informatization (info) is measured by telecom revenue as a share of GDP. The level of informatization is an important factor that can affect the financial network system, improve the urban emergency service management system, measure the national modernization level and promote the resilience of the urban economy.

(7) The urban-rural income gap (inco) is measured by the ratio of urban and rural per capita disposable income. An excessive income gap will lead to the polarization of household consumption, which is not conducive to the diversified development of industries. In addition, the income gap can impact consumption by affecting government transfer payments, which in turn affect urban economic resilience.

#### 3.2.4. Instrumental Variable

(1) Similarity between dialects and Mandarin (diadist). The lower the similarity between the dialect and Mandarin, the worse the Mandarin proficiency of the local labour force. When going out for work, individuals may face language barriers. They may prefer to stay local rather than travel for job opportunities. As a result, the similarity between dialects and Mandarin affects population mobility and is largely unaffected by urban economic resilience. The Beijing dialect is the most similar to Mandarin among all the dialects in the cities. Therefore, “the distance of dialects” from each city to Beijing is used to approximate the degree of similarity between dialects and Mandarin [42]. Dialect categories are divided according to China’s *Linguistics Atlas*. This book divides the dialects of each county into the following three levels: dialect big area, dialect area, as well as dialect piece. The specific assignment rule is as follows: the dialect distance between two countries is 0 if they are in the same dialect piece; If they are in the same dialect area but separate dialect pieces the distance is 1; the dialect distance is 2 if two counties are situated in the same dialect big region but separate dialect areas; and if two counties are located in different dialect big areas, the dialect distance is 3. The specific measurement method of transforming the dialect distance from the county level to the city level is based on Liu’s [42] idea.

(16) $$diadist(A,B)=\sum_{i}^{I}\sum_{j}^{J}S_{Ai}\times S_{Bj}\times d_{ij}$$

where diadist(A,B) represents the dialect distance between city *A* and city *B*, the dialect distance between county *i* and county *j* is $d_{ij}$, $S_{Ai}$ is the proportion of the population of county *i* in city *A* to the total population of city *A*, and $S_{Bj}$ is the proportion of the population of county *j* in city *B* to the total population of city *B*.

(2) Terrain up and down degrees (ter). The study shows that topographic relief can affect population mobility [43]. This paper uses topographic relief as an instrumental variable of population flow. As a topographic element, it does not have the conditions necessary to affect the resilience of the urban economy.

#### 3.2.5. Adjusting Variables

Based on a literature review and mechanism analysis, it can be concluded that population migration may be an important factor to improve urban economic resilience. Furthermore, the research further argues that population mobility affects urban economic resilience and is not homogenous. Because of the differences between the attributes, innovation investment (inno) and fixed asset investment (inv) of cities, the impact degree of urban economic resilience will be different even under the same scale of population migration. Based on this, the number of R&D personnel and per capita fixed asset investment are selected to represent the innovation investment effect and fixed asset investment effect, respectively. Additionally, the path for improving urban economic resilience against the backdrop of population mobility is examined. Table 2 lists the descriptive statistics results for each major variable.

### 3.3. The Fundamental Relationship between Population Mobility and Urban Resilience

**Figure 2:**
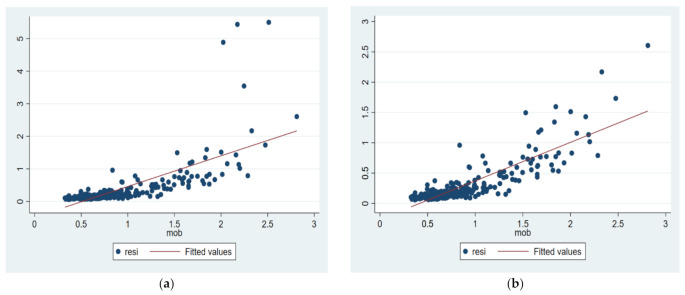
The relationship curve between economic resilience and population mobility. (**a**) Keep extremum, (**b**) Delete extremum.

As the scatter diagram of population flow and urban economic resilience (Figure 2) shows, there is a straight line slanting to the upper right between them. In Figure 2, the value of urban economic resilience increases with the increase of population flow, and there is an obvious positive correlation, which provides strong support for the verification of hypothesis H1 and the corresponding analysis. The four singular values in Figure 2a are Beijing, Shanghai, Shenzhen, Suzhou and other cities, which have strong urban economic resilience. These four singular values were deleted (Figure 2b) to prevent the existence of singular values from influencing the research and interfering with the accuracy of the results, and it was found that the degree of fitting between them was better after deletion. Therefore, these four cities are not taken into account in the empirical analysis in the following paper to overcome possible estimation bias. However, these values will be added again in the robustness test of Section 4.2.4 to verify the robustness of the results.

### 3.4. Model Specification

Based on Formulas (10)–(12) of Section 3.1.4 and the above discussion, we set the spatial econometric model as follows:

(17) $$resi_{i}=\rho Wresi_{i}+\beta_{1}mob_{i}+W\beta_{2}mob_{i}+\beta_{3}{\mathrm{control}}_{i}+W\beta_{4}control_{i}+\mu_{i}$$

(18) $$\mu_{i}=\lambda W\mu_{i}+\varepsilon$$

where *i* represents the city, W represents the spatial weight matrix, and control represents all control variables in Section 3.2.3.

## 4. Analysis of Empirical Results

### 4.1. Spatial Correlation

**Figure 3:**
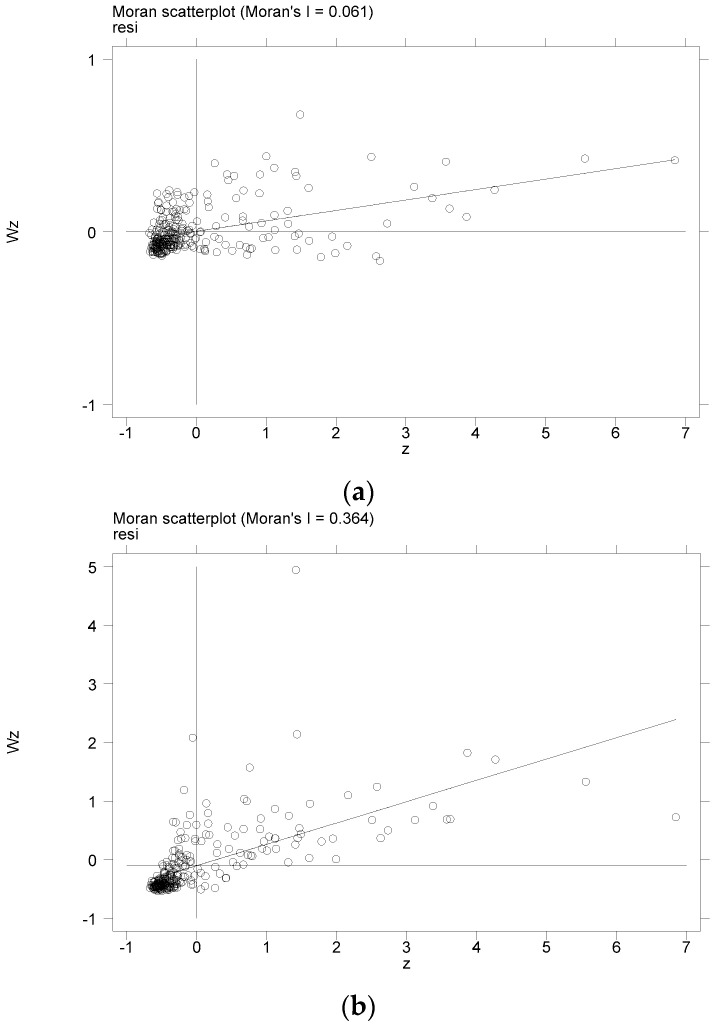

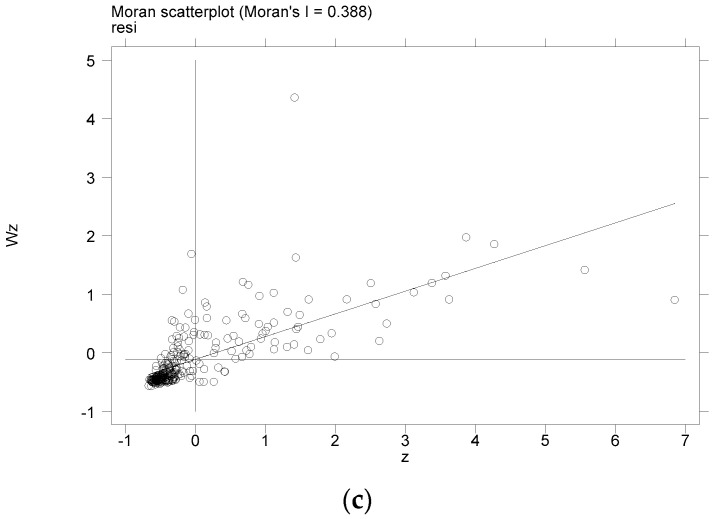
Urban economic resilience of Moran’s I scatterplot. (**a**) W1, (**b**) W2, (**c**) W3.

W1, W2, and W3 refer to the geographic matrix, economic matrix and geo-economic matrix, respectively. In W1, W2, and W3, the global Moran index values of urban economic resilience are 0.061, 0.364 and 0.388, respectively, which are all significant at the 0.01% level (Table 3). The results show that urban economic resilience is spatially correlated, whether geographically adjacent, economically adjacent or geo-economic adjacent. As evidenced by the size of the global Moran’s I coefficient, the spatial correlation of urban economic resilience is the strongest in W3, and the spatial correlation is relatively weak in W2 and W1. The relationship between the Moran scatter plot scatter distribution and trend line matching (Figure 3) indicate that the scattered points in the three matrices are primarily located in the first and third quadrants, showing a positive correlation and obvious degree of autocorrelation, reflecting the geographical neighborhood, economic neighborhood or geo-economics neighborhood cities, and urban economic resilience has high aggregation and low aggregation, that is, the influence is positive.

### 4.2. Spatial Measurement Results and Analysis

#### 4.2.1. Regression of Spatial Reference

Table 4 shows the spatial regression results of population flow and urban economic resilience, including the SAR, SEM and SDM models and the W1, W2 and W3 matrices. The regression findings demonstrate that the estimated coefficients of population mobility are all positive and significant at the 1% level, indicating that cities with more net population inflow have higher levels of urban economic resilience. Columns (1)–(3) report the regression findings of the three weight matrices under the SAR model. The results show that the spatial autocorrelation coefficients of urban economic resilience in the W1, W2 and W3 matrices are all greater than 0 and pass the significance test with a confidence degree of 99%, demonstrating that urban economic resilience has a favourable spatial spillover impact, which can enhance the economic resilience levels of cities with similar distances through spatial and geographical transmission mechanisms and will also be positively affected by cities with similar economic strengths.

Columns (4)–(6) report the regression results of the three weight matrices under the SEM. The findings demonstrate that the spatial error coefficients in the W1, W2 and W3 matrices are all greater than 0 and pass the significance test with a confidence degree of 99%. The findings reveal that although the influence direction of each unobservable component of nearby areas on urban economic resilience varies, these characteristics positively impact urban economic resilience on the whole. At the same time, by comparing the estimated parameter values of population flow in the SEM and SAR variables, the direction of the effect of population flow on urban economic resilience is found to remain unchanged under the influence of unobservable factors, but the impact degree increases. Columns (7)–(9) report the regression results of the three weight matrices under the SDM. The results show that the spatial coefficient of the core explanatory variable population mobility is negative but not significant; the result is consistent with intuition; that is, urban economic resilience under Conditions W1, W2 and W3 will have fallen victim to the population mobility of the neighbourhood. The reasons are as follows: (1) the inflow population may make decisions that cause competition among cities located close to each other; and (2) cities with similar levels of economic development are highly replaceable, and the competition for talent will be more intense, while cities with large disparities in economic development more easily form the population siphoning phenomenon. All of these factors will reduce the economic resilience of cities that are geographically and economically adjacent to another city.

#### 4.2.2. Adding the Spatial Econometric Regression of Instrumental Variables

Given that cities with more resilient economies are better able to cope with external disturbances, with better economic development, the jobs of the floating population are relatively stable, so it is more attractive for outsiders to flow into the local area, which could result in reverse causality. At the same time, there may be a very small measurement error problem with population mobility. These aspects will skew the estimated coefficient of core explanatory variable mob. Therefore, this paper introduces instrumental variables to better reflect the intensity and direction. All regressions adopt robust standard errors to overcome the possible heteroscedasticity problem.

Column (10) of Table 5 shows the simple OLS regression result. The coefficient estimate of population mobility is 0.5241, which passes the significance test with a confidence degree of 99%. The instrumental variables lndiadist and lnter are added to Columns (11) and (12). The regression results of the first stage (Column (11)) show that the estimated coefficient of the instrumental variable lndiadist to the endogenous variable mob is significant at the 5% level, and the coefficient estimates of the instrumental variable lnter to the endogenous variable mob passed the significance test with a confidence level of 99%. The F statistic is 10.381, and the *p* value is 0.0000, which verifies that both instrumental variables have good explanatory power for endogenous variables. In addition, the “LIML” estimation, which is less sensitive to weak instrumental variables, is carried out. The estimated value of the mob coefficient is 0.5120, which is very close to the result of the 2SLS estimation, confirming that there is no weak instrumental variable problem. The *p* value of overidentification is 0.1039, which means that the null hypothesis that all instrumental factors are exogenous cannot be rejected, proving that the instrumental variables are exogenous. The regression results of the second stage (Column (12)) show that, after the addition of instrumental variables, from the direction of coefficients, population mobility significantly improves urban economic resilience. In addition, the regression coefficient of mob decreased, indicating that reverse causality and inevitable measurement errors together led to the OLS results overestimating the impact of migration on urban economic resilience.

Columns (13)–(15) are the spatial regression results after the addition of instrumental variables; that is, on the basis of the regression of instrumental variables, the SDM model is adopted to construct W1, W2 and W3. The results show that the positive correlation between population mobility and urban economic resilience is stable after the endogenous problems and spatial effects are solved, which once again indicates that population mobility will improve urban economic resilience. At this point, H1 is fully verified. In addition. As the coefficient size indicates, compared with the case of W1, W2 and W3, population mobility has a more obvious improvement intensity effect on urban economic resilience.

#### 4.2.3. The Spatial Spillover Test of Instrumental Variables Was Added

The spatial lag term of population mobility is taken into account in the SDM, meaning that the change in population movement affects both the local urban economic resilience and the economic resilience of neighboring regions. If only the regression results of the instrumental variables are used, the spatial feedback effect may be ignored, resulting in deviation. Therefore, referring to the study of Elhorst [44], the partial differential method is adopted to decompose the spillover effect of the SDM, and the direct effect is the influence of population mobility on the local economy’s resilience; the indirect effect is the average influence of population mobility on other nearby regions; and the total effect is the sum of the direct and indirect effects.

The decomposition results of the spatial spillover test are shown in Table 6. Through observation, it can be found that, under the three spatial weight matrices, the direct effect coefficient of mob is highly consistent with the general regression coefficient of the SDM with instrumental variables, which again verifies the robustness of the results. In matrix W2 and matrix W3, the indirect effects are 0.6546 and 0.7530, respectively. The total effects are 1.2328 and 1.3410, respectively, indicating that the net inflow of population in local cities promotes the economic resilience of adjacent cities, and this effect exists between cities with similar economic attributes or between geo-economic adjacent cities. In matrix W1, the indirect effects were −0.8170 and −0.4539, which did not pass the significance test, but the result was intuitive. Due to geographical proximity, the net inflow of the population caused conflicts among cities; that is, part of the inflow of the population in the region comes from the outflow of the population from nearby cities. Therefore, the impact of population flow on the urban economic resilience of geographically adjacent cities is negative.

#### 4.2.4. Robustness Test

Figure 2a indicates that the strong urban economic resilience of Beijing, Shanghai, Shenzhen and Suzhou may interfere with the empirical results. To reduce estimation bias, they were removed from the preceding empirical analysis. This section includes the four cities with excessively strong economic resilience in the benchmark regression as a robustness test, and the results are as follows. Table 7 reports the robust regression results in the case of W1, W2 and W3. Columns (16)–(18) use the SDM, and the results show that the coefficient of mob is still significantly positive, which supports the basic conclusion of this paper, i.e., population mobility will improve urban economic resilience. In addition, by comparing the regression results with Table 5, it is found that the coefficient of mob is greatly increased in matrices W1 and W3, while the coefficient of mob is significantly decreased in matrix W2. This indicates that the addition of these four cities will have an excessive impact on the population mobility to urban economic resilience regression results and interfere with the final coefficient estimation.

## 5. Analysis of the Regulation Mechanism

According to the above analysis, urban economic resilience is promoted by population movement, but we still do not understand the regulatory mechanism. Therefore, this paper provides further discussion. From the analysis of 2.3 and 2.4, it can be observed that population mobility may affect urban economic resilience through innovation input and fixed asset investment. Therefore, the SDM is selected to construct the regulating effect, and the adjustment mechanism of population mobility on urban economic resilience is investigated from these two aspects. The interaction variables were decentralized to avoid the impact of multicollinearity on the results and ensure the validity and consistency of the model estimation results. Meanwhile, the VIF test, carried out for all explanatory variables, was approximately 2.23, providing no multicollinearity problem.

### 5.1. Model Setup

Based on Models (17) and (18), the following adjustment model is constructed by adding the cross term of innovation input, fixed asset investment, innovation input and population flow, as well as the cross term of fixed asset investment and population flow, as follows:

(19) $$\begin{array}{ll} resi= & \rho Wresi_{i}+\beta_{1}mob_{i}+W\beta_{2}mob_{i}+\beta_{3}\ln inno+\beta_{4}mob\ln inno+\\ & W\beta_{5}\ln inno+W\beta_{6}mob\ln inno+\beta_{7}control_{i}+W\beta_{8}control_{i}+\mu_{i} \end{array}$$

(20) $$\begin{array}{ll} resi= & \rho Wresi_{i}+\beta_{1}mob_{i}+W\beta_{2}mob_{i}+\beta_{3}\ln inno+\beta_{4}mob\ln inv+\\ & W\beta_{5}\ln inv+W\beta_{6}mob\ln inv+\beta_{7}control_{i}+W\beta_{8}control_{i}+\mu_{i} \end{array}$$

(21) $$\mu =\lambda W\mu_{i}+\varepsilon$$

### 5.2. Moderating Effect Test

#### 5.2.1. Innovation Input Effect

Table 8 reports the regulating effects of innovation investment and fixed asset investment in the SDM. Columns (19)–(21) of Table 8 analyze the regulatory effect of innovation input on the relationship between population mobility and urban economic resilience under the W1, W2 and W3 matrices. According to the regression results, at the 1% level of significance, the coefficient value of population flow and the interaction term (moblninno) coefficient of innovation input and population flow are both positive. The three matrices convey highly consistent information, and the results are robust. This indicates that innovation input has a positive moderating effect between them. With the increase of innovation input and the improvement of innovation level, in cities with strong innovation vitality, population mobility plays a stronger role in promoting urban economic resilience. The reasons may lie in the following two aspects: First, scientific and technological innovation can promote the optimization and upgrading of urban industrial structure, accelerate the birth of new economic production activities, and thus form a new path of economic growth to cope with external shocks. In this case, the enhancement of inter-city population flow will greatly improve the resilience of the urban economy. Secondly, technological innovation can not only liberate rural productivity, but also promote the transfer of the rural surplus labor force to the city. Moreover, it can lead to the emergence of a series of emerging industries, provide a large number of employment opportunities, and improve the city’s ability to absorb the rural surplus labor force. At this time, the flow of population will play a more obvious role in promoting the resilience of the urban economy. By comparing the results of the regression coefficients in Table 5 and Table 8, it can be determined that innovation input is the mechanism path for population mobility to promote urban economic resilience. Hypothesis H2 is confirmed. According to the spatial spillover effect, in W1 and W2, the enhancement of the local innovation input effect may weaken the moderating effect of innovation input in population mobility on urban economic resilience, resulting in a “beggar-thy-neighbor” competition situation. The possible reason is that areas with a more significant innovation input effect will have higher labor utilization efficiency, and the siphon effect is more likely to occur between nearby cities, which will attract labor resources or other resources from surrounding cities to gather in the local city, thus weakening the regulatory effect of innovation input in surrounding cities. In other words, cities with similar economic development levels may compete with each other in terms of the innovation input effect, and labor and other resources may tend to flow into cities with stronger innovation input effects, thus weakening the moderating effect of innovation input in population mobility on urban economic resilience. However, for W3, this competitive situation is not significant.

#### 5.2.2. Fixed Asset Investment Effect

Columns (22)–(24) of Table 8 analyze the regulatory effect of fixed asset investment in population mobility on urban economic resilience under the W1, W2 and W3 matrices. At the 1% level of significance, the coefficient value of population flow and the interaction term (moblninv) coefficient of innovation input and population flow are both positive according to the regression results. The three matrices continue to convey highly consistent information, and the results are robust. This indicates that fixed asset investment has a positive moderating effect between them. With the increase of fixed asset investment, the city has great potential for future economic development and relatively high-quality employment. Stable employment guarantees people’s income and provides huge space for urban economic development. In this case, the increase of the inter-city population flow will greatly improve the resilience of the urban economy. In addition, fixed asset investment plays a guiding role in the optimization of industrial structure and can promote the upgrading of it. The continuous optimization of industrial structure makes the urban economic structure more reasonable. Therefore, the large flow of population will have a more obvious promoting effect on the resilience of the urban economy. The results of the regression coefficients in Table 5 and Table 8 show that the effects of fixed asset investment exist in the process of population mobility, enhancing the resilience of the urban economy, and Hypothesis H3 is confirmed. According to the spatial spillover effects, in the W2 and W3 matrices, the enhancement of the regulating effect of investment in fixed assets in this region will enhance the moderating effect of fixed asset investment in neighboring areas on the urban economic resilience of population flow. This shows that fixed asset investment will not only promote the vitality of local economic resilience but also that the cities that are economically adjacent or geo-economic adjacent could both benefit, showing significant positive externalities. However, this positive externality is not significant in the case of W1, the possible reason is that in most areas of China, the construction of urban agglomeration and urban circle is not mature, and the radiation driving effect on the surrounding areas has a certain effect, but it is not obvious. It is necessary to strengthen the degree of connection and correlation among cities within the urban agglomeration and further optimize the allocation of various economic factors within the region.

## 6. Conclusions and Suggestions

### 6.1. Conclusions

This paper discusses the mechanism by which population mobility affects urban economic resilience based on Baidu migration big data. The results indicate the following:

(1) Urban economic resilience is spatially correlated in W1, W2 and W3, and there are high-high aggregations and low-low aggregations in space, and the correlation order is W3 > W2 > W1.

(2) The economic resilience levels of inflow areas are significantly influenced positively by the population net inflow. By introducing the similarity between dialects and Mandarin and the terrain up and down degree, a more accurate result is found, that is, the urban economic resilience index increases by 0.36–0.56% when the population mobility index increases by one unit.

(3) Through the decomposition of spatial spillover effects, it can be concluded that in the case of economic adjacency and geo-economic adjacency, there is a spatial interaction relationship of “building a good partnership with its neighbors” in the effect mechanism of population migration on urban economic resilience.

(4) Innovation input has a favorable moderating influence on urban economic resilience and population mobility. With the increase in innovation input, the promotion effect of population mobility on urban economic resilience will be enhanced. According to the spatial spillover effect, in W1 and W2, the innovation input effect has a negative externality.

(5) Fixed asset investment has a favorable moderating influence on urban economic resilience and population mobility. With the increase in fixed asset investment, the promotion effect of population mobility on urban economic resilience will be enhanced. According to the spatial spillover effect, the fixed asset investment effect has a significant positive externality in the case of W2 and W3.

### 6.2. Suggestions

First, we make use of spatial correlation to play the driving role of economic resources between cities. According to the research, we find that urban economic resilience is spatially correlated, and the spatial autocorrelation test shows that there are phenomena of high-high and low-low concentrations of urban economic resilience. That is, the interactions between different cities have some positive feedback or some low-level negative effects. This research argues that to construct strong urban economic resilience, on the one hand, we need to fully utilize the typical leadership role of pilot demonstration, strengthen the mobility of the value of economic resources between cities, generate spatial effects such as cooperation, learning, imitation and competition through externalities, capital and personnel flows, and information technology sharing, and encourage individual cities to take the lead in setting an example and make breakthroughs with the help of cities with high levels of economic resilience to promote the economic resilience of surrounding cities. On the other hand, it is necessary to be vigilant in resisting the negative impact of urban economic resilience on neighboring municipalities. Interactions between cities are not always positive and are sometimes negative. For example, cities with low levels of economic resilience are also less able to respond to risks and challenges, which not only hinders their own economic development but also hampers the economic development power of surrounding cities. In short, the spatial distribution of urban economic resilience is characterized by imbalanced and inadequate levels. To prevent this imbalanced and inadequate effect from evolving into a serious spatial Matthew effect, the driving effect of high levels of urban economic resilience on the economic resources of surrounding cities should be given special consideration.

Second, population mobility should be reasonably promoted between cities. According to the research, we find that the economic resilience of inflow areas is significantly positively influenced by the net inflow of the population. Although population mobility may bring problems such as rising housing prices [45] and urban diseases [46] to urban economic development, at present, the continuous inflow of population has always been proven to be an indispensable driving force for creating a better economic environment [47]. The premium power should be constantly enhanced. First, first-tier cities and central cities are the main areas of population inflow. At the present stage, population control cannot be used to solve the urban economic development problem. In contrast, it is necessary to maintain a relatively open population migration policy, transform the conditions for settling down and relax the restrictions on settling down. Additionally, an inclusive system should be developed to provide a friendly social environment for migrants who are unwilling or unable to settle down. Second, for cities with small population inflows and large population outflows, the population concentration of these cities is low, and industrial development is often limited by labor shortages. This part of the region should not only remove restrictions on settlement but also, more importantly, actively develop a series of incentive policies, provide employment opportunities, improve the quality of employment, improve public facilities and services, and improve the living environment and quality of life, which will increase the attractiveness of these cities to the population, achieve balanced population mobility, guarantee regional economic vitality, and enhance urban economic resilience. Finally, population flow should be properly controlled in light of the current situation of COVID-19 prevention and control. The common measures used to prevent the spread of COVID-19 are lockdown and quarantine, which, while helping to stop the further spread of COVID-19, will inevitably aggravate the impact of the epidemic on economic development [48,49]. However, if we allow excessive population movement, it will put enormous pressure on the epidemic prevention and control work, and also hinder the improvement of economic resilience. Therefore, the government needs to adopt corresponding policies and supporting measures according to the specific situation and the city’s current resource endowment and economic foundation to jointly promote the population flow management during the epidemic, which can not only ensure the public health of the masses, but also generally not hinder the urban economic development.

Third, cities should increase investment in innovation. The results show that innovation input can enhance the promoting effect of population mobility on urban economic resilience, which is an important moderating variable. In addition, the innovation input effect may be competitive between cities that are geographically close or have similar levels of urban economic development. Therefore, local governments need to do the following three things. (1) They should adopt an innovation-driven strategy and increase investment in innovation, especially for the training of innovative talent. The essence of innovation is that it is talent-driven, and local governments should focus on key areas, cultivate talent with special characteristics, serve urban development, and convert human capital into talent capital. We will adopt an innovation-driven strategy and improve the talent security mechanism. We will increase social security for migrant workers in cities, expand the coverage of basic services, and provide opportunities for the floating population and their family members. We need to avoid the competition between cities that leads to the flow of well-trained talent to geographic or economic proximity not only to attract people, but also to retain people. (2) The efficiency of innovation resource utilization should be improved, the innovation environment should be continuously optimized, human resources should be reasonably allocated, and human capital should be consolidated. Local governments should focus on the matching and coordination degree of innovation and human capital in different stages of urban areas and adjust and optimize innovation resources and talent policies dynamically in real time so that the accumulation of human resources and innovation development levels of cities at the current stage can develop in a coordinated coupling. The loss of economic factors due to the misallocation of innovation resources and the idleness of human capital should be avoided. (3) Focus on the practical application of innovation. Especially in the context of COVID-19, innovation is the key to solving the conflict between population mobility, public health and economic development. For example, big data analysis technology has played an important role in close contact tracing and epidemic prediction [50]. Under the normal movement of people, it has reduced the risk of epidemic transmission, ensured the public health and safety of the people, and enhanced the resilience of the urban economy.

Fourth, they should focus on the scientific regulation of fixed asset investment. The findings show that enhancing effective investment in fixed assets significantly enhances the contribution of population mobility to urban economic resilience, and furthermore, the fixed asset investment effect has significant positive externalities between cities with economic proximity and geo-economic proximity. Therefore, the reconciliation effect of fixed assets should be utilized with maximum effectiveness. Specific paths include (1) improving the efficiency of fixed asset investment. The Fourteenth Five-Year Plan clearly stated the goal to “adhere to the development of economic focus on the real economy”, which cannot be separated from investment. However, it should be noted that investment here refers to effective investment, not to reduce the economic efficiency of repeated investment. Because of the government’s limited resources, it is necessary to continuously optimize the regional allocation structure of investment and build an investment mechanism with multiple channels to maximize the efficiency of fixed asset investment. (2) A more reasonable growth rate of fixed asset investment should be maintained. There are still structural factors supporting fixed asset investment in China; that is, the human advantage has not been fully utilized in the central and western areas, where there are many idle labor resources. In view of this situation, boosting fixed asset investment is still an efficient approach to stimulate economic growth and assist the central and western regions in their ascent. (3) Give full play to the driving force of government investment. The government should scientifically regulate fixed asset investment, drive industrial development and optimize industrial structure through effective investment in fixed assets so that the labor force flowing into the city can be fully utilized to achieve the maximum promotion effect of population mobility on urban economic resilience and make full use of the positive externality of wielding the effect of fixed asset investment to drive the improvement of the economic resilience of cities in surrounding areas.

But it should be noted that the higher mobility of the population will also bring some disadvantages to urban development. For example, in some eastern regions with high population density, high mobility and aggregation degree, the urban land carrying space tends to be saturated, and high population inflow will bring great pressure on the infrastructure and public services that are already in short supply, which is not conducive to the improvement of urban economic resilience. On the contrary, labor is already scarce in some areas of the central and western regions, in addition to northeast China, and the massive population outflow has aggravated the labor shortage, which further slows the economic development which is already lacking in power, and is not conducive to enhancing the resilience of the urban economy. In addition, population flow may further complicate government management. Due to its complex composition, large number and frequent change frequency, the floating population will increase the management difficulties and costs of the government. In terms of social effect, the surge of urban population flow will bring great challenges to community security and social security, meanwhile accompanied by a large number of “left-behind children”, “empty-nesters”, as well as urban ecological resource consumption and environmental pollution [51]. In terms of public health, China’s population flow is large and complicated, and most of these people have weak health awareness. Large-scale migration will increase the chance of disease transmission and pose a serious threat to public health, especially in the context of the ongoing COVID-19 pandemic. Based on this, we should carefully weigh the advantages and disadvantages brought by the mobility of the urban population and work out the most suitable strategic planning for the current development of the city.

## Figures and Tables

**Table 1:** Urban Economic Resilience Index Syste.

	Criterion Layer	Index Layer	Weights
Economic resilience	Crisis resistance	GDP per capita	0.0540
		Import and export trade volume	0.3889
		The proportion of the output value of the secondary and tertiary industries in GDP	0.0064
	Urban resilience	Per capita RMB deposit balance of financial institutions	0.1395
		Per capita general public budget revenue	0.1430
		Proportion of employees in the tertiary industry	0.0155
	Risk transformation	Spending on science and education as a percentage of GDP	0.0521
		Number of college students per 10,000 people	0.0747
		Per capita total retail sales of consumer goods	0.1260

Note: The weights in the table are calculated according to the entropy method.

**Table 2:** Descriptive statistics.

Variable	Definition	Unit	Mean	Sd	Min	Max
resi	Economic resilience	1	0.348	0.639	0.0584	5.501
mob	Population mobility	1	0.883	0.478	0.325	2.812
lnscal	Urban size	10,000	5.897	0.715	3.045	8.134
open	Economic openness	%	1.328	1.525	0.00112	8.689
fina	Financial environment	1	1.525	1.460	0.161	19.56
gov	Government management	%	7.550	2.436	2.343	18.78
lninfr	Infrastructure construction	m^2^/person	1.698	0.665	0.243	3.993
info	Level of information	%	2.304	3.129	0.399	18.65
inco	Urban-rural income gap	1	2.238	0.395	1.290	3.402
lndiadist	Dialect distance	-	1.195	0.151	0.219	1.386
lnter	Terrain	-	0.442	0.386	0	1.898
lninno	Innovation input	person	8.772	1.745	2.639	13.05
lninv	Investment scale	CNY	10.62	0.734	5.996	12.07

**Table 3:** Urban economic resilience global Moran’s I.

W	I	E (I)	Sd (I)	Z	*p* Value
W1	0.061	−0.004	0.005	12.865	0.000
W2	0.364	−0.004	0.029	12.640	0.000
W3	0.388	−0.004	0.030	13.155	0.000

**Table 4:** Economic resilience and population mobility: baseline regression.

	(1)	(2)	(3)	(4)	(5)	(6)	(7)	(8)	(9)
	resi	resi	resi	resi	resi	resi	resi	resi	resi
	SAR			SEM			SDM		
	W1	W2	W3	W1	W2	W3	W1	W2	W3
mob	0.4804 ***	0.4746 ***	0.4525 ***	0.5096 ***	0.5105 ***	0.5000 ***	0.4590 ***	0.4441 ***	0.4211 ***
	(0.0302)	(0.0319)	(0.0330)	(0.0299)	(0.0295)	(0.0299)	(0.0328)	(0.0308)	(0.0326)
lnscal	0.0980 ***	0.0987 ***	0.0988 ***	0.0984 ***	0.1013 ***	0.1020 ***	0.1008 ***	0.1074 ***	0.1055 ***
	(0.0149)	(0.0151)	(0.0149)	(0.0157)	(0.0152)	(0.0154)	(0.0170)	(0.0141)	(0.0146)
open	0.0041	0.0040	0.0040	0.0047	0.0058	0.0042	0.0011	0.0018	0.0015
	(0.0066)	(0.0067)	(0.0066)	(0.0069)	(0.0067)	(0.0067)	(0.0072)	(0.0064)	(0.0065)
fina	0.0186 ***	0.0168 **	0.0171 ***	0.0175 ***	0.0151 **	0.0154 **	0.0200 ***	0.0145 **	0.0156 **
	(0.0065)	(0.0066)	(0.0065)	(0.0066)	(0.0066)	(0.0065)	(0.0065)	(0.0061)	(0.0062)
gov	0.0075 *	0.0088 **	0.0085 **	0.0073 *	0.0088 **	0.0086 **	0.0070 *	0.0099 ***	0.0099 ***
	(0.0039)	(0.0040)	(0.0039)	(0.0040)	(0.0039)	(0.0039)	(0.0040)	(0.0037)	(0.0037)
lninfr	0.1227 ***	0.0981 ***	0.0930 ***	0.1230 ***	0.1202 ***	0.1270 ***	0.1356 ***	0.1000 ***	0.1044 ***
	(0.0213)	(0.0220)	(0.0218)	(0.0219)	(0.0216)	(0.0216)	(0.0216)	(0.0213)	(0.0215)
info	0.0052 *	0.0059 *	0.0060 **	0.0052 *	0.0043	0.0040	0.0048	0.0035	0.0048 *
	(0.0030)	(0.0031)	(0.0030)	(0.0031)	(0.0030)	(0.0030)	(0.0031)	(0.0029)	(0.0029)
inco	0.0138	−0.0216	−0.0148	−0.0187	−0.0271	−0.0268	0.0231	−0.0189	−0.0028
	(0.0264)	(0.0248)	(0.0246)	(0.0266)	(0.0245)	(0.0253)	(0.0312)	(0.0235)	(0.0249)
ρ	1.2162 ***	0.2601 ***	0.3653 ***				1.6220	0.6523 ***	0.9120 ***
	(0.2744)	(0.0714)	(0.0840)				(1.0909)	(0.1790)	(0.2166)
λ				0.9694 **	0.2015 **	0.3590 ***			
				(0.4023)	(0.0958)	(0.1219)			
W*mob							−0.1590	−0.0738	−0.2102
							(0.9021)	(0.1451)	(0.1611)
W*lnscal							−0.4709 ***	−0.1794 ***	−0.0804 ***
							(0.1686)	(0.0388)	(0.0307)
W*open							0.0568	0.0143	0.0217
							(0.0716)	(0.0193)	(0.0172)
W*fina							0.0350	−0.0005	−0.0043
							(0.1225)	(0.0290)	(0.0209)
W*gov							−0.0054	−0.0384 ***	−0.0207 *
							(0.0713)	(0.0114)	(0.0114)
W*lninfr							−0.8339 ***	−0.2025 ***	−0.1699 ***
							(0.2550)	(0.0562)	(0.0528)
W*info							−0.0923 *	0.0031	0.0070
							(0.0485)	(0.0080)	(0.0085)
W*inco							−0.0323	−0.1309 *	−0.0363
							(0.2827)	(0.0708)	(0.0653)
N	283	283	283	283	283	283	283	283	283
Pesduo.R2	0.7820	0.7783	0.7762	0.7757	0.7760	0.7757	0.8012	0.8164	0.8160

Note: Standard errors in parentheses, * *p* < 0.1, ** *p* < 0.05, *** *p* < 0.01, W*X represents the spatial term of the explanatory variable.

**Table 5:** Results of instrumental variable estimation.

	(10)	(11)	(12)	(13)	(14)	(15)
	OLS	First Stage	IV	W1	W2	W3
mob	0.5241 ***		0.5140 ***	0.3594 ***	0.5508 ***	0.5556 ***
	(0.0508)		(0.1052)	(0.0902)	(0.0852)	(0.0973)
lnscal	0.0987 ***	0.1737 ***	0.1002 ***	0.1207 ***	0.0952 ***	0.0910 ***
	(0.0189)	(0.0366)	(0.0207)	(0.0238)	(0.0168)	(0.0182)
open	0.0062	0.0065	0.0062	0.0029	0.0043	0.0044
	(0.0076)	(0.0135)	(0.0075)	(0.0075)	(0.0067)	(0.0065)
fina	0.0161 ***	−0.0029	0.0160 ***	0.0194 ***	0.0149 **	0.0163 ***
	(0.0033)	(0.0107)	(0.0032)	(0.0066)	(0.0062)	(0.0063)
gov	0.0082	0.0046	0.0083	0.0074 *	0.0093 **	0.0089 **
	(0.0068)	(0.0085)	(0.0068)	(0.0040)	(0.0038)	(0.0038)
lninfr	0.1146 ***	0.5072 ***	0.1196 **	0.1776 ***	0.0621 *	0.0617 *
	(0.0297)	(0.0374)	(0.0541)	(0.0416)	(0.0329)	(0.0332)
info	0.0049	0.0024	0.0050	0.0051 *	0.0027	0.0038
	(0.0033)	(0.0052)	(0.0031)	(0.0031)	(0.0030)	(0.0030)
inco	−0.0318	−0.1271 **	−0.0321	0.0214	−0.0194	−0.0064
	(0.0225)	(0.0556)	(0.0226)	(0.0318)	(0.0239)	(0.0255)
lndiadist		0.3006 **				
		(0.1522)				
lnter		0.2417 ***				
		(0.0621)				
ρ				1.8191 **	0.5550 ***	0.5885 ***
				(0.7365)	(0.1195)	(0.1469)
W*lnscal				−0.5993 ***	−0.1657 ***	−0.0602 *
				(0.2005)	(0.0424)	(0.0345)
W*open				0.0606	0.0181	0.0316 *
				(0.0687)	(0.0196)	(0.0178)
W*fina				0.0388	0.0047	0.0014
				(0.1303)	(0.0286)	(0.0211)
W*gov				−0.0308	−0.0361 ***	−0.0192
				(0.0732)	(0.0120)	(0.0118)
W*lninfr				−0.9331 ***	−0.2532 ***	−0.2462 ***
				(0.2418)	(0.0577)	(0.0543)
W*info				−0.0849 *	0.0027	0.0047
				(0.0500)	(0.0081)	(0.0088)
W*inco				0.0100	−0.1144	−0.0332
				(0.3070)	(0.0711)	(0.0654)
N	283	283	283	283	283	283
adj. R2	0.770	0.501	0.770			
Pesduo.R2				0.7924	0.8152	0.8141

Note: Standard errors in parentheses, * *p* < 0.1, ** *p* < 0.05, *** *p* < 0.01, W*X represents the spatial term of the explanatory variable.

**Table 6:** Spillover effect of decomposition results.

W	Variable	Direct Effect	Indirect Effect	Total Effect
W1	mob	0.3631 ***	−0.8170	−0.4539
W2	mob	0.5782 ***	0.6546 ***	1.2328 ***
W3	mob	0.5880 ***	0.7530 ***	1.3410 ***

Note: *** *p* < 0.01.

**Table 7:** Economic resilience and population mobility: join the extremum.

	(16)	(17)	(18)
	resi	resi	resi
	W1	W2	W3
Mob	1.2663 ***	0.3867	0.8553 ***
	(0.2681)	(0.2563)	(0.2447)
Lnscal	0.0750	0.2251 ***	0.1623 ***
	(0.0730)	(0.0517)	(0.0526)
Open	−0.0015	0.0017	0.0056
	(0.0217)	(0.0195)	(0.0185)
Fina	0.0378 **	0.0277	0.0308 *
	(0.0189)	(0.0182)	(0.0177)
Gov	0.0087	0.0137	0.0106
	(0.0114)	(0.0109)	(0.0108)
Lninfr	−0.1369	0.1803 *	0.0109
	(0.1254)	(0.1023)	(0.0928)
Info	0.0019	0.0074	0.0040
	(0.0090)	(0.0089)	(0.0087)
Inco	0.0443	−0.0338	−0.0075
	(0.0923)	(0.0693)	(0.0726)
ρ	−0.2725	0.9678 **	0.5745 **
	(1.2503)	(0.3901)	(0.2338)
W*explanatory	YES	YES	YES
N	287	287	287
Pesduo.R2	0.5250	0.4526	0.5658

Note: (1) Standard errors in parentheses, * *p* < 0.1, ** *p* < 0.05, *** *p* < 0.01, (2) W*explanatory represents the spatial term of the explanatory variable.

**Table 8:** The existence test results of innovation input and fixed asset investment effect.

	(19)	(20)	(21)	(22)	(23)	(24)
	resi	resi	resi	resi	resi	resi
	W1	W2	W3	W1	W2	W3
mob	0.1292 ***	0.1517 ***	0.1219 ***	0.3785 ***	0.3769 ***	0.3669 ***
	(0.0410)	(0.0382)	(0.0389)	(0.0382)	(0.0336)	(0.0345)
lnscal	0.0355 *	0.0396 **	0.0434 **	0.0924 ***	0.0976 ***	0.1009 ***
	(0.0194)	(0.0179)	(0.0177)	(0.0167)	(0.0134)	(0.0137)
open	0.0038	−0.0027	0.0008	−0.0006	0.0010	0.0006
	(0.0061)	(0.0056)	(0.0055)	(0.0071)	(0.0061)	(0.0061)
fina	0.0184 ***	0.0172 ***	0.0166 ***	0.0168 ***	0.0108 *	0.0125 **
	(0.0054)	(0.0053)	(0.0051)	(0.0063)	(0.0058)	(0.0057)
gov	0.0042	0.0051	0.0050	0.0064 *	0.0076 **	0.0080 **
	(0.0033)	(0.0032)	(0.0031)	(0.0039)	(0.0035)	(0.0034)
lninfr	0.1393 ***	0.1093 ***	0.1102 ***	0.1349 ***	0.1055 ***	0.1136 ***
	(0.0198)	(0.0192)	(0.0184)	(0.0218)	(0.0199)	(0.0201)
info	0.0037	0.0045 *	0.0056 **	0.0024	0.0012	0.0026
	(0.0026)	(0.0025)	(0.0024)	(0.0030)	(0.0027)	(0.0027)
inco	0.0135	0.0099	0.0282	0.0233	−0.0218	−0.0003
	(0.0264)	(0.0204)	(0.0206)	(0.0309)	(0.0221)	(0.0233)
lninno	0.0317 ***	0.0242 ***	0.0224 ***			
	(0.0092)	(0.0084)	(0.0084)			
moblninno	0.1620 ***	0.1434 ***	0.1517 ***			
	(0.0143)	(0.0141)	(0.0137)			
lninv				0.0173	0.0123	0.0042
				(0.0180)	(0.0156)	(0.0160)
moblninv				0.1636 ***	0.1192 ***	0.1086 ***
				(0.0417)	(0.0411)	(0.0410)
ρ	4.1097 **	0.9309 ***	0.6005 **	2.2557 *	0.4076 **	0.5086 **
	(1.6462)	(0.2614)	(0.2768)	(1.2602)	(0.1850)	(0.2014)
W*mob	0.8473	0.0783	0.1066	−0.0135	−0.2222	−0.2741 *
	(0.7185)	(0.1175)	(0.1133)	(0.8496)	(0.1566)	(0.1474)
W*lnscal	−0.2623	−0.0870 *	−0.0292	−0.3757 **	−0.1648 ***	−0.1434 ***
	(0.2298)	(0.0494)	(0.0400)	(0.1738)	(0.0404)	(0.0374)
W*open	−0.0250	0.0061	0.0198	0.0702	0.0283	0.0194
	(0.0953)	(0.0178)	(0.0158)	(0.0808)	(0.0188)	(0.0169)
W*fina	−0.0274	−0.0232	−0.0108	0.0148	−0.0386	−0.0249
	(0.1032)	(0.0247)	(0.0176)	(0.1248)	(0.0277)	(0.0200)
W*gov	0.0226	−0.0277 ***	−0.0176 *	0.0385	−0.0306 ***	−0.0242 **
	(0.0596)	(0.0098)	(0.0095)	(0.0787)	(0.0109)	(0.0107)
W*lninfr	−1.1170 ***	−0.1433 **	−0.0672	−0.8215 ***	−0.1039 *	−0.1255 **
	(0.3227)	(0.0618)	(0.0527)	(0.2876)	(0.0571)	(0.0545)
W*info	−0.0948 **	0.0004	0.0028	−0.0784	−0.0076	−0.0043
	(0.0453)	(0.0069)	(0.0072)	(0.0492)	(0.0077)	(0.0080)
W*inco	0.0099	−0.1269 **	−0.0873 *	0.1409	−0.0994	−0.0944
	(0.2433)	(0.0599)	(0.0525)	(0.3098)	(0.0682)	(0.0603)
W*lninno	−0.1722	−0.0428	−0.0303			
	(0.1617)	(0.0273)	(0.0246)			
W*moblninno	−1.2396 ***	−0.1873 **	−0.0830			
	(0.4213)	(0.0753)	(0.0742)			
W*lninv				−0.1769	0.0135	0.0714 **
				(0.1590)	(0.0333)	(0.0291)
W*moblninv				−1.0918	0.4636 ***	0.4945 ***
				(0.9651)	(0.0940)	(0.0952)
N	283	283	283	283	283	283
Pesduo.R2	0.6934	0.8233	0.8759	0.7820	0.8422	0.8440

Note: Standard errors in parentheses, * *p* < 0.1, ** *p* < 0.05, *** *p* < 0.01.

## Data Availability

The data presented in this study are openly available in the China. Statistical Yearbook, China City Statistical Yearbook, China Statistical Yearbook for Regional Economy, EPS database, statistical yearbooks of relevant provinces and cities, statistical bulletins of national economic, social development and Baidu Map migration big data platform.
